# Intestinal Calculus from a Horse

**Published:** 1883-07

**Authors:** 


### XII.—INTESTINAL CALCULUS FROM A HORSE.

REPORTED FROM DR. WALTON'S CLINIC.

The patient was a large bay horse used for draft work. The horse was first taken sick February, 1883. The symptoms at that time were those of ordinary colic, but they developed and increased with unusual rapidity. The case was at first treated for an ordinary colic, but instead of improving he steadily grew worse. The second day of the attack, the animal being so bad was brought to the hospital, and from that time was under constant observation.

For three days there was no movement of the bowels, although Oleum Tiglii and all forms of drastic cathartics were used in conjunction with enemas.

Treatment, however, proved of no avail, and on the sixth day the horse died without a positive diagnosis having been made, further than some form of intestinal obstruction. At the necropsy the true cause of this obstinate trouble was elucidated. A large calculus was found in the colon about the size of a man's head, and as hard as a stone. It could hardly be broken with a hammer. The other organs showed marked congestion, but otherwise were normal.

